# Supplementary material for: An essential role for Argonaute 2 in EGFR-KRAS signaling in pancreatic cancer development
Source: Nat Commun. 2020 Jun 4;11:2817. doi: 10.1038/s41467-020-16309-2 (PMC7272436; doi:10.1038/s41467-020-16309-2)
Supplement: Supplementary file 3 — Reporting Summary [file 41467_2020_16309_MOESM3_ESM.pdf]

nature research

Corresponding author(s): Arul M. Chinnaiyan

Last updated by author(s): Apr 8, 2020

Reporting Summary

Nature Research wishes to improve the reproducibility of the work that we publish. This form provides structure for consistency and transparency in reporting. For further information on Nature Research policies, see Authors & Referees and the Editorial Policy Checklist.

Statistics

For all statistical analyses, confirm that the following items are present in the figure legend, table legend, main text, or Methods section.

n/a	Confirmed	
☐	☒	The exact sample size (*n*) for each experimental group/condition, given as a discrete number and unit of measurement
☐	☒	A statement on whether measurements were taken from distinct samples or whether the same sample was measured repeatedly
☐	☒	The statistical test(s) used AND whether they are one- or two-sided<br>*Only common tests should be described solely by name; describe more complex techniques in the Methods section.*
☒	☐	A description of all covariates tested
☒	☐	A description of any assumptions or corrections, such as tests of normality and adjustment for multiple comparisons
☐	☒	A full description of the statistical parameters including central tendency (e.g. means) or other basic estimates (e.g. regression coefficient) AND variation (e.g. standard deviation) or associated estimates of uncertainty (e.g. confidence intervals)
☐	☒	For null hypothesis testing, the test statistic (e.g. *F*, *t*, *r*) with confidence intervals, effect sizes, degrees of freedom and *P* value noted<br>*Give P values as exact values whenever suitable.*
☒	☐	For Bayesian analysis, information on the choice of priors and Markov chain Monte Carlo settings
☒	☐	For hierarchical and complex designs, identification of the appropriate level for tests and full reporting of outcomes
☒	☐	Estimates of effect sizes (e.g. Cohen's *d*, Pearson's *r*), indicating how they were calculated

Our web collection on statistics for biologists contains articles on many of the points above.

Software and code

Policy information about availability of computer code

Data collection	Details of microRNA qPCR data and transcriptome analysis provided in Methods section
Data analysis	Log-transformed miRNA measurements performed using GenEx software ver6.<br>IMAJEJ (ImageJ-win64) was downloaded from https://imagej.net/Fiji/Downloads<br>edgeR_3.28.0<br>limma_3.42.0<br>Rsubread_2.0.0 (featureCounts)<br>RSeQC-2.6.4<br>STAR-2.7.3a

For manuscripts utilizing custom algorithms or software that are central to the research but not yet described in published literature, software must be made available to editors/reviewers. We strongly encourage code deposition in a community repository (e.g. GitHub). See the Nature Research guidelines for submitting code & software for further information.

Data

Policy information about availability of data

All manuscripts must include a data availability statement. This statement should provide the following information, where applicable:

- Accession codes, unique identifiers, or web links for publicly available datasets
- A list of figures that have associated raw data
- A description of any restrictions on data availability

All data generated or analyzed during this study are included in this article. This includes raw data for the immunoblot (Main Fig. 2c; 6c, e, f, g, h; 7a, b, c, d, e, f, g, h, i; 8a, b, c, d; and Supplementary Fig. 2; 7b, c; 12a; 15a, b, c) and microRNA analyses (Fig. 6a, Supplementary Fig. 10a, b). A reporting summary for this article is available as a Supplementary Information file. The RNA sequencing data (for Fig. 6b and Supplementary Fig. 11) have been deposited in GEO under the accession

number GSE147781.

Field-specific reporting

Please select the one below that is the best fit for your research. If you are not sure, read the appropriate sections before making your selection.

☒ Life sciences ☐ Behavioural & social sciences ☐ Ecological, evolutionary & environmental sciences

For a reference copy of the document with all sections, see nature.com/documents/nr-reporting-summary-flat.pdf

Life sciences study design

All studies must disclose on these points even when the disclosure is negative.

Sample size	Using tools available at http://www.biomath.info/power/ttest.htm, it was determined that less than 6 animals would yield statistical significant results for the mouse model. In our study, we have used at least 12 animals in each group.
Data exclusions	No data were excluded.
Replication	Reproducibility of results were ensured by 1) involving multiple members of the team to collect data, 2) analyzing pathologies with two independent pathologists, and 3) repeating cell line-based experiments at least twice. Many experiments were repeated by multiple members of the group.<br>The data are representative of multiple biological replicates, and the number of times each of the individual data was repeated with similar results is indicated below.<br>Main Figures-<br>Two: 5c,5f,6d,6e,6f,7g,7h,7j, 8c,8d<br>Three: 1b,2c,2d, 5a,5g,6g,6h, 7a,7b,7d,7i,8a, 8b<br>Four: 2f,7c,7e, 7f<br>Six: 6i,6j,6k<br>Eight: 3c<br>Ten: 4a,1c,5b,5d,5e<br>Also note that for Fig.2a., this image is representative of data collected in Fig. 2b and 4b: PanIn:6, PDAC: 6, metastases: 2<br>Supplementary Figures-<br>Two: 1a, 2, 7b, 7c, 7d, 8b, 8c, 8d,13c, 14c, 15a, 15b, 15c, 16a, 16b<br>Three: 7e,9a, 14a, 14b<br>Four: 6,9c, 13a<br>Six: 3a, 4a, 5, 8a<br>Micrographs shown in 4b represent each of the abnormal pathologies observed.
Randomization	Since experimental animals were genetically engineered and had similar backgrounds, randomization was not applicable to the study design.
Blinding	Blinding was performed for pathological assessment. Also, while set up of pathological experiments itself was not blinded, data analysis was blinded in all cases.

Reporting for specific materials, systems and methods

We require information from authors about some types of materials, experimental systems and methods used in many studies. Here, indicate whether each material, system or method listed is relevant to your study. If you are not sure if a list item applies to your research, read the appropriate section before selecting a response.

Materials & experimental systems

n/a	Involved in the study	
☐	☒	Antibodies
☐	☒	Eukaryotic cell lines
☒	☐	Palaeontology
☐	☒	Animals and other organisms
☐	☒	Human research participants
☒	☐	Clinical data

Methods

n/a	Involved in the study	
☒	☐	ChIP-seq
☒	☐	Flow cytometry
☒	☐	MRI-based neuroimaging

Antibodies

Antibodies used	Antibody dilutions used are detailed in Supplementary Table 1.<br>Anti-Ras clone 10 (RAS10) Millipore 05-516<br>K-Ras-2B Antibody (C-19) Santa Cruz sc-521<br>K-Ras monoclonal Ab Santa Cruz sc-30

RAS (G12D Mutant Specific)
DH87 Cell Signaling 14429S
KRAS (3B10-2F2) Sigma- aldrich WH0003845M1
HRAS- specific Antibody Proteintech 18295-1-AP
AGO2, 11A9 Sigma SAB4200085
AGO2 EIF2C2 Sino Biologicals 50683-R036
Anti- EGFR (phospho Y1092) Abcam ab40815 (EP774Y)
Phospho-EGF Receptor (Y1068) Cell Signaling 2234S
Phospho-p44/42 MAPK (Erk 1/2) Cell Signaling 4376
ERK1(K-23) Santa Cruz sc-94
Total p44/42 MAPK (Erk 1/2) Cell Signaling 9102
EGFR Antibody Abcam ab52894 (EP38Y)
EGFR(1005) Santa Cruz sc-03
Anti-FLAG antibody Sigma F7425
Phospho-Akt S473 Cell Signaling 4060S
AKT (pan) - C67E7 Cell Signaling 4691
Anti-Cytokeratin 19 antibody Abcam ab133496
E-cadherin (36) Ventana Roche 790-4497
Nk1.1 (PK136) Invitrogen MA1-70100
anti-CD4 (SP35) Ventana 790-4423
anti-CD68 (KP-1) Ventana 790-2931
anti-CD8 (SP57) Ventana 790-4460
Anti-Cytokeratin 19 antibody Abcam ab133496
GAPDH- HRP Cell Signaling 3683
Normal mouse IgG Santa Cruz sc-2025
Normal Rat IgG Abcam ab18450
Normal Rabbit IgG Millipore 12-370

Validation

Validation of the antibodies used in the study:
References and links cited below show specificity and use of the respective antibody for various applications.
1. RAS antibodies (Anti-Ras clone 10 (RAS10), K-Ras-2B Antibody (C-19), K-Ras monoclonal Ab, RAS (G12D Mutant Specific) ,DH87 KRAS (3B10-2F2), HRAS- specific antibodies have been validated (1).) Manufacturer's instruction provides evidence for use of RAS10 in IHC/IF. This is in addition to the extensive validation performed in this study. For all other antibodies, further details are available at the manufacturer's website.
2. AGO2, 11A9 (SAB4200085) https://www.sigmaaldrich.com/catalog/product/mm/mabe253?lang=en&region=US
3. AGO2 Sino biological (50683-R036) https://www.sinobiological.com/antibodies/mouse-argonaute-2-50683-r036
4. Anti- EGFR (phospho Y1092) ab40815 (EP774Y) https://www.abcam.com/egfr-phospho-y1068-antibody-ep774y-ab40815.html
and confirmed in house by immunoblot analysis; also referenced here (2).
5. Phospho-EGF Receptor (Y1068) CST 2234S https://www.cellsignal.com/products/primary-antibodies/phospho-egf-receptor-tyr1068-antibody/2234
6. Phospho-p44/42 MAPK (Erk 1/2) CST 4376 https://www.cellsignal.com/products/primary-antibodies/phospho-p44-42-mapk-erk1-2-thr202-tyr204-d13-14-4e-xp-rabbit-mab/4370?site-search-type=Products
7. ERK1(K-23) sc-94 https://www.scbt.com/p/erk-1-antibody-k-23; also referenced in (3).
8. Total p44/42 MAPK (Erk 1/2) CST 9102 https://www.cellsignal.com/products/primary-antibodies/p44-42-mapk-erk1-2-antibody/9102
9. EGFR Antibody ab52894 (EP38Y) https://www.abcam.com/egfr-antibody-ep38y-ab52894.html In-house validation showed that the IHC pattern of expression for this antibody was identical to EFGR 1005 sc-03. Recognizes single band of correct molecular weight in mouse tissues as well.
10. EGFR(1005) sc-03 https://www.scbt.com/p/egfr-antibody-1005
11. Anti-FLAG antibody Sigma F7425 https://www.sigmaaldrich.com/catalog/product/sigma/f7425?lang=en&region=US
12. Phospho-Akt CST 4060S https://www.cellsignal.com/products/primary-antibodies/phospho-akt-ser473-d9e-xp-rabbit-mab/4060
13. AKT (pan) - C67E7 4691 https://www.cellsignal.com/products/primary-antibodies/akt-pan-c67e7-rabbit-mab/4691
14. Anti-Cytokeratin 19 antibody ab133496 https://www.abcam.com/cytokeratin-19-antibody-epncir127b-ab133496.html
15. E-cadherin (36) Ventana Roche 790-4497 http://reagent-catalog.roche.com/product/1518?type=1958
16. Nk1.1 (PK136) Invitrogen MA1-70100 https://www.thermofisher.com/antibody/product/NK1-1-Antibody-clone-PK136-Monoclonal/MA1-70100
17. anti-CD4 (SP35) Ventana 790-4423 http://reagent-catalog.roche.com/product/31?type=26
18. anti-CD68 (KP-1) Ventana 790-2931http://reagent-catalog.roche.com/product/53?type=48
19. anti-CD8 (SP57) Ventana 790-4460 http://reagent-catalog.roche.com/product/33?type=28
20. GAPDH- HRP CST 3683 https://www.cellsignal.com/products/antibody-conjugates/gapdh-14c10-rabbit-mab-hrp-conjugate/3683
21. Normal mouse IgG sc-2025 https://www.scbt.com/p/normal-mouse-igg
22. Normal Rat IgG Abcam ab18450 https://www.abcam.com/rat-igg2a-kappa-monoclonal-rtk2758-isotype-control-low-endotoxin-azide-free-ab18450.html
23. Normal Rabbit IgG Millipore 12-370https://www.emdmillipore.com/US/en/product/Normal-Rabbit-IgG,MM_NF-12-370

References
1. Waters, A.M., et al. Evaluation of the selectivity and sensitivity of isoform- and mutation-specific RAS antibodies. Sci Signal 10(2017).
2. Wodziak, D., Dong, A., Basin, M.F. & Lowe, A.W. Anterior Gradient 2 (AGR2) Induced Epidermal Growth Factor Receptor (EGFR) Signaling Is Essential for Murine Pancreatitis-Associated Tissue Regeneration. PLoS One 11, e0164968 (2016).
3. Navas, C., et al. EGF receptor signaling is essential for k-ras oncogene-driven pancreatic ductal adenocarcinoma. Cancer Cell

22, 318-330 (2012).

Eukaryotic cell lines

Policy information about cell lines

Cell line source(s)	PC3 (ATCC)<br>MCF7 (ATCC)<br>A375 (ATCC)<br>A549 (ATCC)<br>MIA PaCa-2 (ATCC)<br>CAPAN-1 (ATCC)<br>DLD-1 (MUT/WT) (ATCC)<br>H358 (ATCC)<br>HCT116 (ATCC)<br>PANC-1 (ATCC)<br>Panc 05.04 (ATCC)<br>Panc 10.05 (ATCC)<br>HEK293FT (ATCC)<br>MEF,parental (Zamore lab (PMID: 21878547))<br>AGO2 MEF-/- (Zamore lab (PMID: 21878547))<br>AGO2 MEF-/-+AGO2 (Zamore lab (PMID: 21878547))<br>Rasless MEFs (The RAS Initiative https://www.cancer.gov/research/key-initiatives/ras/outreach/reference-reagents)
Authentication	Cell lines were genotyped using STR analysis. Profiles were compared to those provided by ATCC for authentication.
Mycoplasma contamination	All cell lines were tested for mycoplasma every two weeks while maintained in culture. Only cells that were mycoplasma-free were used for analyses.
Commonly misidentified lines (See ICLAC register)	No commonly misidentified cell lines were used for this study.

Animals and other organisms

Policy information about studies involving animals; ARRIVE guidelines recommended for reporting animal research

Laboratory animals	SL-KRASG12D7 (Kras LSL-G12D/+) and p48Cre54 (Ptf1a-Cre or Ptf1aCre/+) mice were obtained from Marina Pasca di Magliano, University of Michigan. Conditionally floxed AGO213 (AGO2fl/fl) mice were purchased from Jackson labs (Bar Harbor, Maine). Given that mice were maintained on a mixed background, littermate controls were systematically used in all experiments (sex ratio per cohort was balanced). All animals were housed in a pathogen-free environment, and all procedures were performed in accordance with requirements of the University of Michigan IACUC.
Wild animals	No wild animals were used in the study.
Field-collected samples	No field-collected samples were used in the study.
Ethics oversight	All animals were housed in a pathogen-free environment, and all procedures were performed in accordance with requirements of the University of Michigan IACUC.

Note that full information on the approval of the study protocol must also be provided in the manuscript.

Human research participants

Policy information about studies involving human research participants

Population characteristics	Patients with pancreas resections for pancreatitis, cystic neoplasms, or PDA from 2002 to 2015 at the University of Michigan Health System were included in the study. All pathology slides were reviewed and diagnosis confirmed by a gastrointestinal pathologist. The electronic medical record was examined for clinical and demographic patient information. Date of surgery and date of last patient contact were recorded from the electronic medical record. Deaths were confirmed from the Social Security Death Index. Clinical staging was analyzed using the American Joint Committee on Cancer 8th edition staging system. For patients who received neoadjuvant treatment, clinical stage was analyzed based on pre-treatment tumor size, while pathological parameters of tumor size, grade, lymph node status, and peripancreatic, duodenal and common bile duct extension were analyzed based on the post-treatment surgical specimen.
Recruitment	Patients with pancreas resections for pancreatitis, cystic neoplasms, or PDA from 2002 to 2015 at the University of Michigan Health System were included in the study.
Ethics oversight	The Institutional Review Board at the University of Michigan approved the study (protocol number: HUM00098128).

Note that full information on the approval of the study protocol must also be provided in the manuscript.
