# Supplementary material for: An essential role for Argonaute 2 in EGFR-KRAS signaling in pancreatic cancer development
Source: Nat Commun. 2020 Jun 4;11:2817. doi: 10.1038/s41467-020-16309-2 (PMC7272436; doi:10.1038/s41467-020-16309-2)
Supplement: Supplementary file 1 — Supplementary Information [file 41467_2020_16309_MOESM1_ESM.pdf]

SUPPLEMENTARY INFORMATION

An Essential Role for *Argonaute 2* in EGFR-KRAS Signaling in Pancreatic Cancer Development

Shankar et.al.

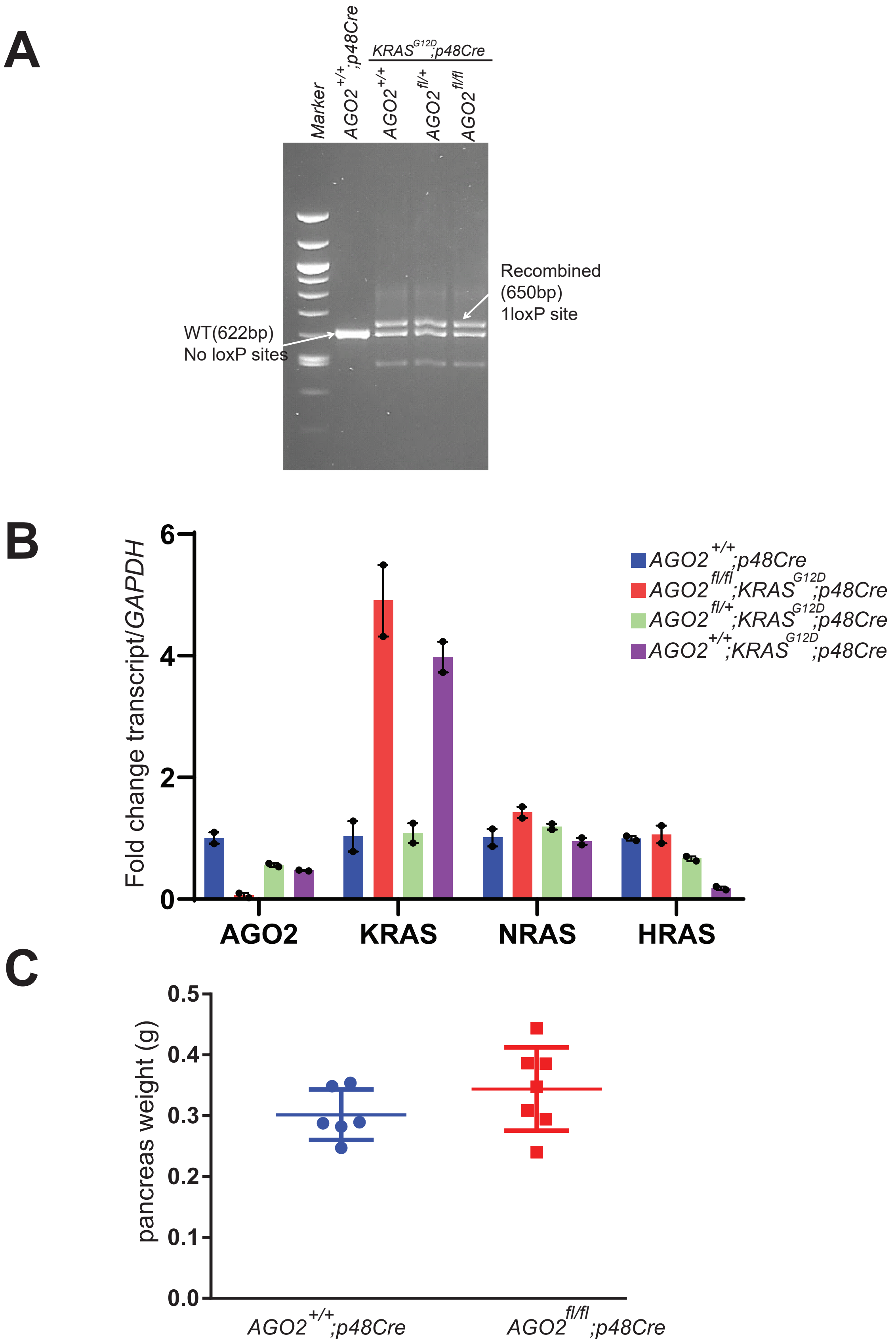

Supplementary Figure 1: **Cre activation in the pancreatic cancer mouse model.** (A) Genomic DNA analysis from the pancreata of the indicated genotypes showing recombined alleles in the LSL-*KRAS* model. (B) RT-qPCR analysis for different transcripts from one 10-week old mouse pancreata of each of the indicated genotypes. Further validations of AGO2 knockout efficiency were performed using immunoblot/immunofluorescence (IF). Error bars indicate mean values +/- SEM of technical replicates. (C) Scatter plot showing the weight of pancreata obtained from six *AGO2*$^{+/+}$;*KRAS*^{G12D};*Cre* and seven *AGO2*$^{fl/fl}$;*KRAS*^{G12D};*Cre* mice of the indicated genotypes at 12 weeks of age. Data are presented as mean values +/- SEM.

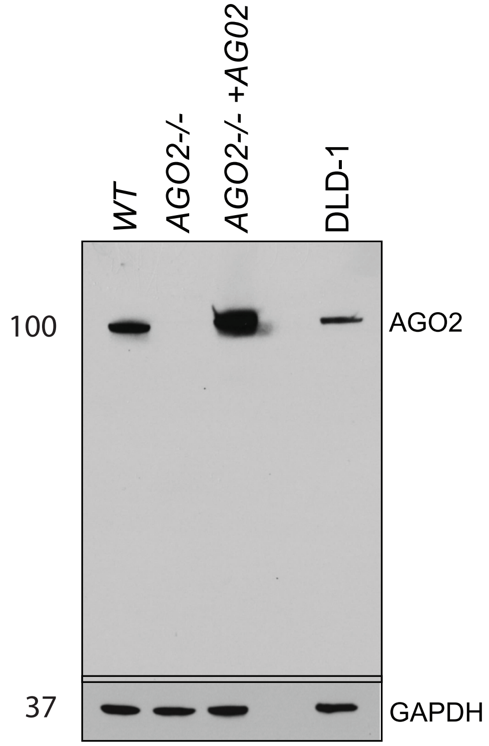

Supplementary Figure 2: **Specificity of AGO2 monoclonal antibody.** Western blot analysis of AGO2-/- mouse embryonic fibroblasts and human colon cancer cells using a monoclonal antibody to AGO2 to confirm specificity of the antibody prior to use in immunohistochemistry (IHC) and IF. Further details are provided in **Supplementary Table 1**. Numbers on the left indicate molecular weight in kDa.

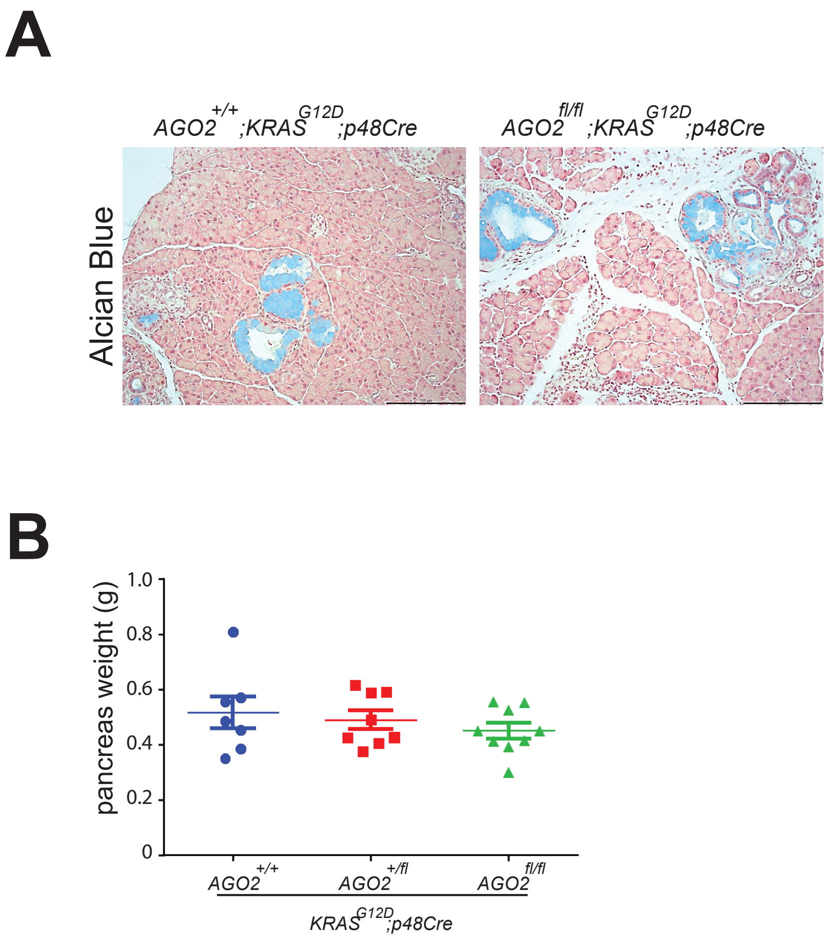

Supplementary Figure 3: **PanINs observed in *AGO2$^{+/+}$;KRAS^{G12D};p48Cre* and *AGO2$^{fl/fl}$;KRAS^{G12D};p48Cre* are similar.** (A) Alcian Blue (mucin) staining of PanINs of the indicated genotypes. Scale bar represents 100 µm. (B) Scatter plot showing the weight of pancreata obtained from eight mice of *Ago2$^{+/+}$;Kras^{G12D};p48Cre,* 20 of *Ago2$^{fl/+}$;Kras^{G12D};p48Cre*, and 13 of *Ago2$^{fl/fl}$;Kras^{G12D};p48Cre* genotype at 12 weeks of age. Data are presented as mean values +/- SEM.

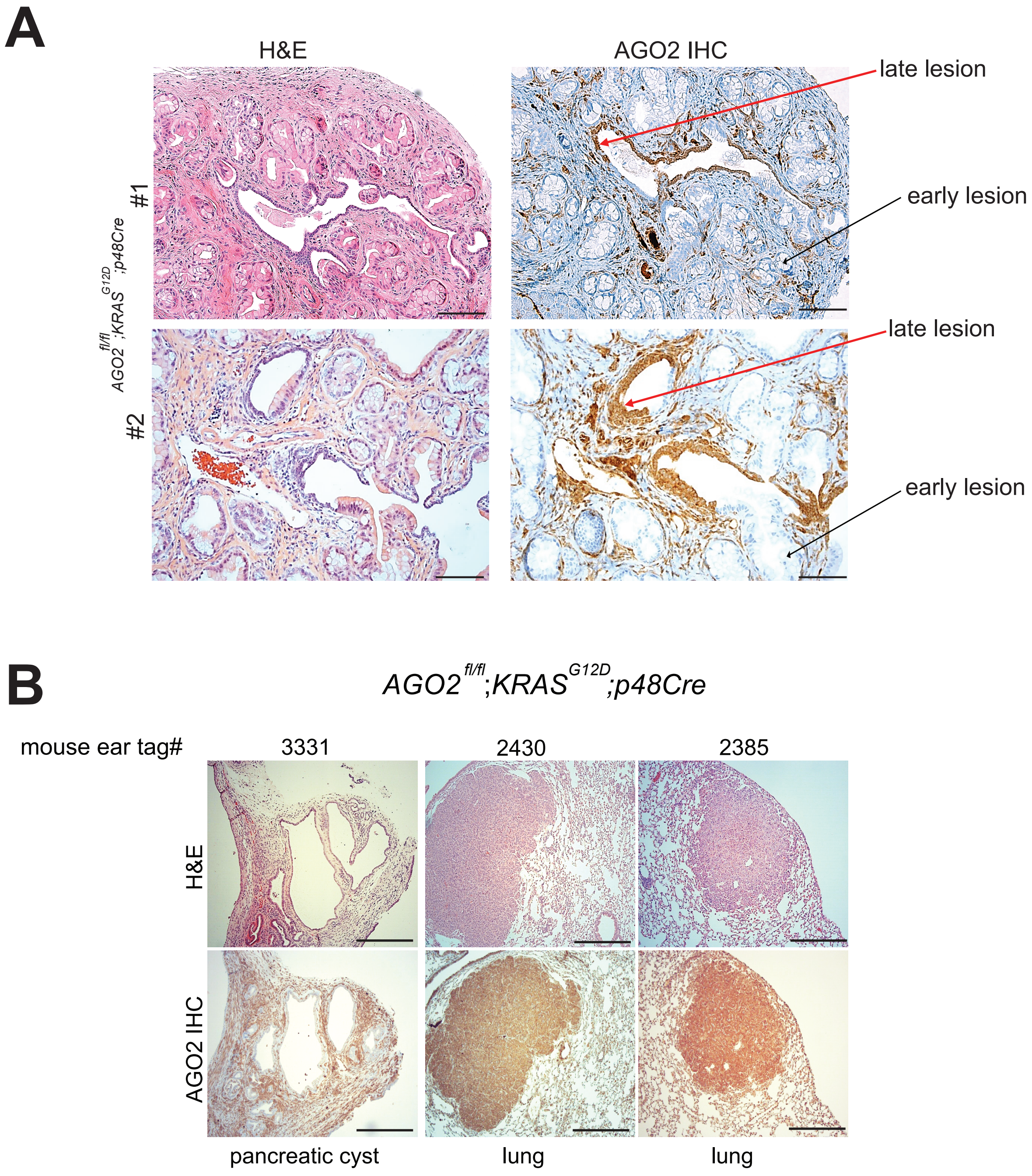

Supplementary Figure 4: Leaky expression and abnormal pathologies in pancreatic tissue with *AGO2* ablation. (A) Representative images of H&E and AGO2 IHC from two animals of the *AGO2*$^{fl/fl}$;*KRAS*^{G12D};*p48Cre* cohort showing AGO2 expression in the late lesions. Scale bar represents 100 µm and 40 µm for upper and lower panels, respectively. (B) H&E and AGO2 IHC analysis of abnormal pancreas and lungs from the *AGO2*$^{fl/fl}$;*KRAS*^{G12D};*p48Cre* mouse cohort (further detailed in **Supplementary Table 2**). Scale bar, 100 µm and 40 µm for low and high magnification, respectively.

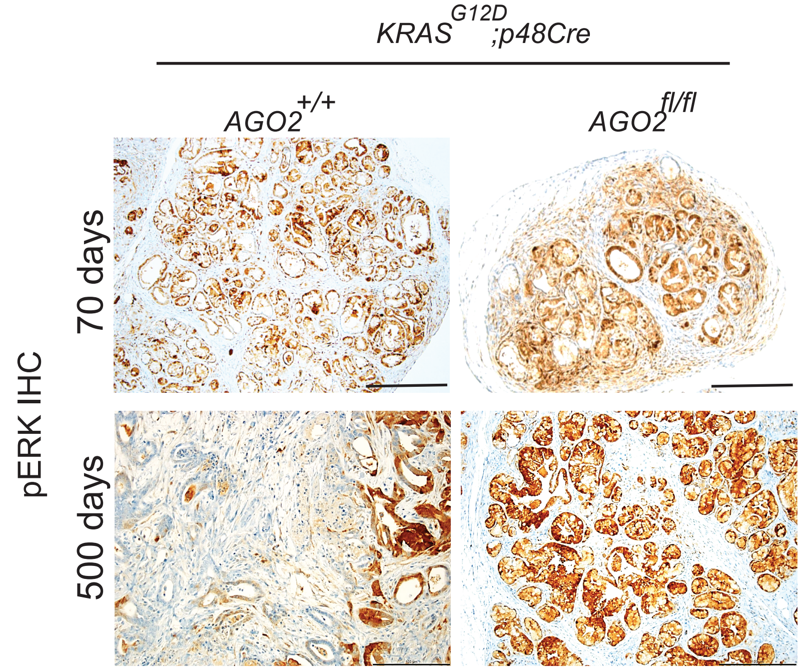

Supplementary Figure 5: High phospho-ERK levels in PanINs of *AGO2*$^{fl/fl}$*;KRAS*^{G12D}*;p48Cre* mice. Representative images of phospho-ERK IHC staining of pancreatic tissues from the indicated genotypes during PDAC progression. Scale bar is 100 µm. Note the inconsistent staining pattern of phospho-ERK in PDAC tissue of *AGO2*$^{+/+}$*; KRAS*^{G12D}*;p48Cre* genotype at 500-day time point.

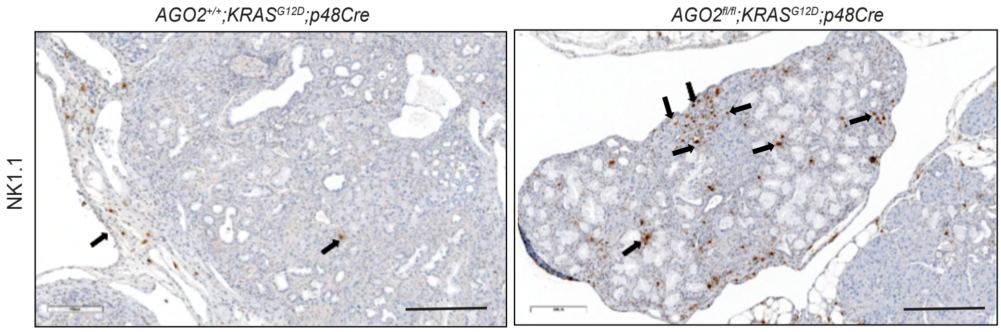

Supplementary Figure 6: Increased NK cell infiltration in the PanIN lesions of *AGO2$^{fl/fl}$;KRAS^{G12D};p48Cre* mice compared to PanINs of *AGO2$^{+/+}$; KRAS^{G12D};p48Cre*. Scale bar is 100 µm.

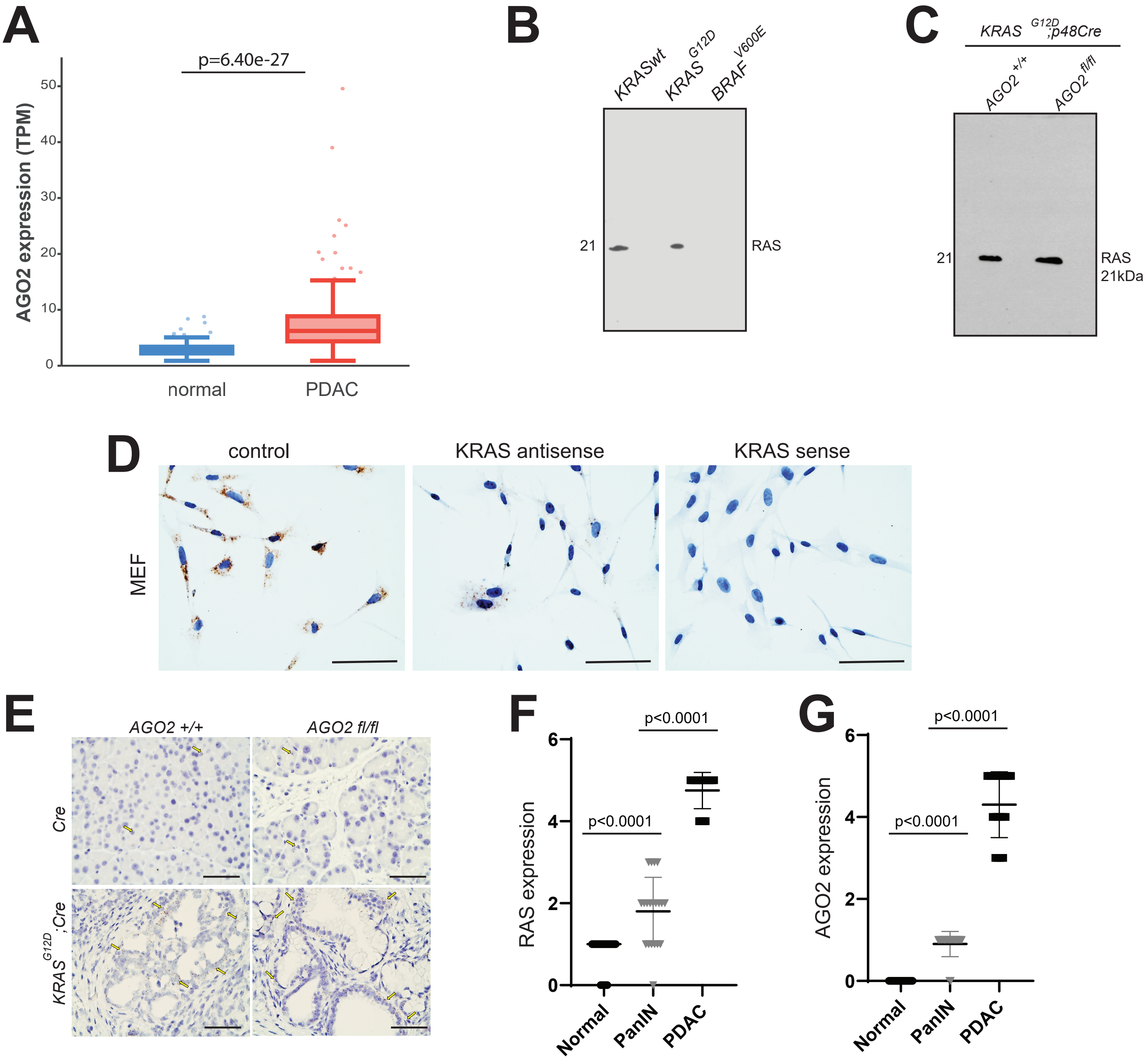

Supplementary Figure 7: Specificity of RAS10 Mab and KRAS/RAS/AGO2 expression in various models. (A) Levels of *AGO2* mRNA transcripts in pancreatic adenocarcinoma in the TCGA dataset compared to levels in normal pancreatic tissue (obtained from GTEX dataset https://gtexportal.org/home). Transcripts from 198 normal and 178 human pancreatic cancer tissues are represented. The minima, median, maxima for normal are 0.657, 2.503, 8.567 and for pancreatic cancer are 1.151, 6.494, 49.839, respectively. The lower and upper quartile for normal are 1.9845 and 3.17075 and for cancer, 4.63675 and 9.11525, respectively. P-value was determined using two sided t-test. TPM: Transcripts per kilobase million. Specificity of the RAS10 antibody confirmed by immunoblot analysis of Rasless MEFs rescued using various KRAS constructs (B) and mouse pancreas expressing oncogenic KRAS (C). Numbers on the left of the blots in (B) and (C) are molecular weights in kDa. Note that complete blots are shown to demonstrate the presence of a single band at 21kDa. Further details are provided in **Supplementary Table 1**. (D) MEFs were assessed for *KRAS* RNA expression using RNA ISH. *KRAS* antisense and sense probes were used as positive and negative controls, respectively. Scale bar represents 50 µm. (E) Representative images of *KRAS* RNA-ISH on pancreatic tissues from normal and $AGO2^{fl/fl}$ animals (top panels) and oncogenic KRAS-expressing $AGO2^{+/+}$;$KRAS^{G12D}$;*Cre* and $AGO2^{fl/fl}$;$KRAS^{G12D}$;*Cre* mice (lower panels). Scale bar, 50 µm. (F) Box plot quantitating RAS or (G) AGO2 expression levels in PanINs and PDAC. Three individual mice were used and 20 PanINs/PDAC were assessed. For (F) and (G), expression values range from 0-5, where 0 is for no expression and 5 is for maximum expression. Unpaired two sided t-test was performed to determine the p-values and data are presented as mean values +/- SEM.

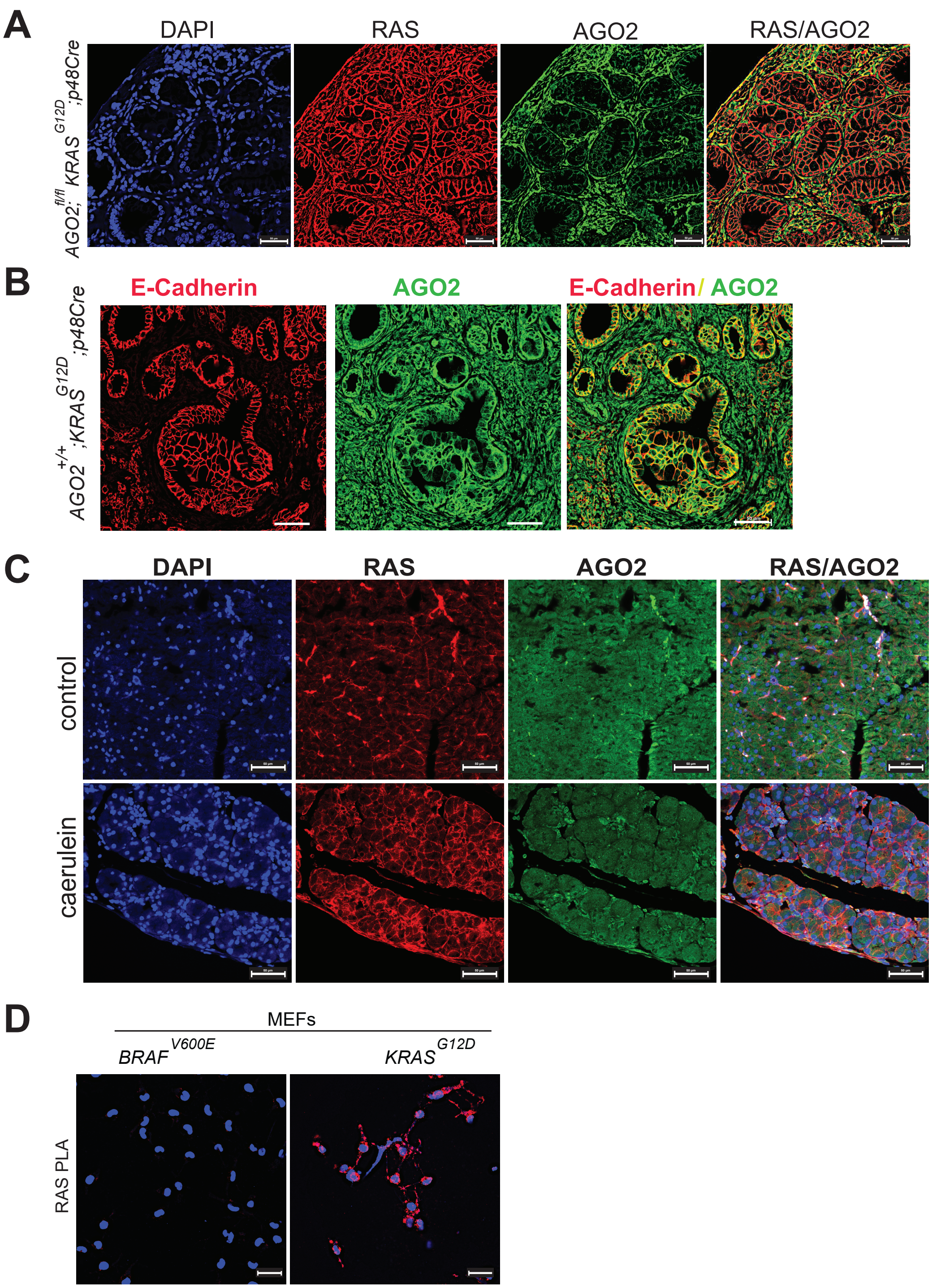

Supplementary Figure 8: IF and proximity ligation assay (PLA) using RAS and AGO2 antibodies. (A) RAS IF staining in *AGO2*$^{fl/fl}$;*KRAS*^{G12D};*Cre* PanINs at 400 days. Scale bar, 50 μm. (B) Membrane localization of AGO2 demonstrated by AGO2/E-Cadherin co-localization signals in early PanIN lesions of *AGO2*$^{+/+}$;*KRAS*^{G12D};*Cre* mice. E-Cadherin is used as a plasma membrane marker. Scale bar, 50 μm. (C) Representative images of RAS and AGO2 IF staining on pancreatic tissue from normal mice after treatment with caerulein to induce pancreatitis. Scale bar, 50 μm. (D) Validation of RAS10 antibody for RAS PLA detection in RASless MEFs expressing either *KRAS*^{G12D} or *BRAF*^{V600E}. Scale bar, 50 μm.

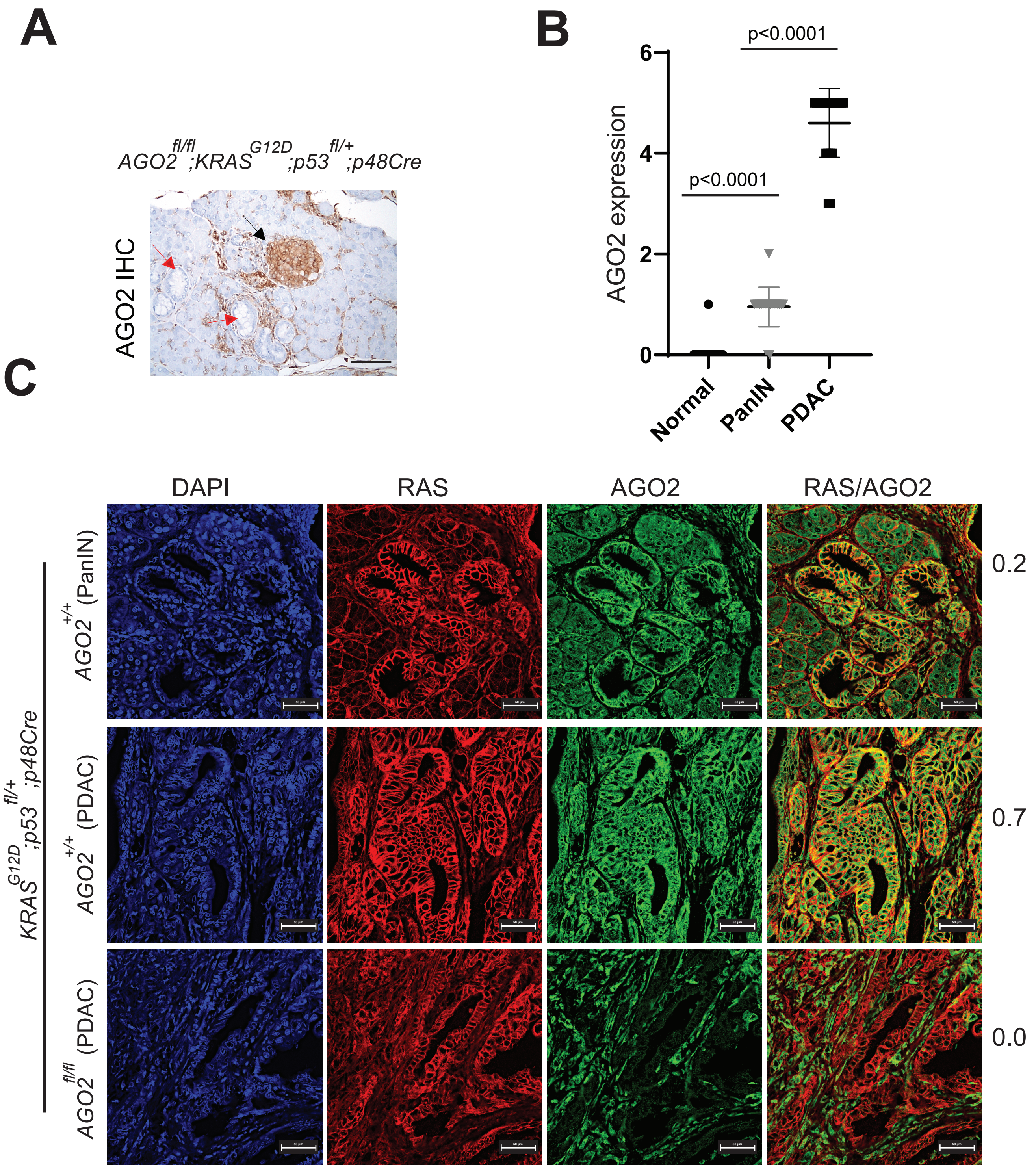

Supplementary Figure 9: IHC and IF staining of RAS/AGO2 in the pancreatic tissue of the KPC model. (A) AGO2 IHC in pancreatic tissue of *Ago2*$^{fl/fl}$*;Kras*^{G12D}*;Trp53*$^{fl/+}$*;p48Cre*. Red and black arrows indicate the PanIN lesions and the islets of Langerhans. Scale bar, 50 µm. (B) Box plot quantitating AGO2 expression levels in PanINs and PDAC. Pancreatic tissue from three individual mice and 20 PanINs/PDAC lesions were assessed. Expression values range from 0-5, where 0 is for no expression and 5 is for maximum expression. Unpaired two sided t-test was performed to determine the p-value. Data are presented as mean values +/- SEM. (C) Representative images of IF analysis for RAS and AGO2 through PDAC progression in the *AGO2*$^{+/+}$*;KRAS*^{G12D}*; Trp53*$^{fl/+}$*;p48Cre* mice. Numbers adjacent to merged images indicate the Pearson's coefficient of co-localization (PCC) of RAS-AGO2 signals at the membranous regions (where 0 is no overlap and 1 is complete overlap). PCC was determined using co-localization signals of at least 50 cells in three distinct areas representative of normal acinar, PanIN, or PDAC. Scale bar, 50 µm.

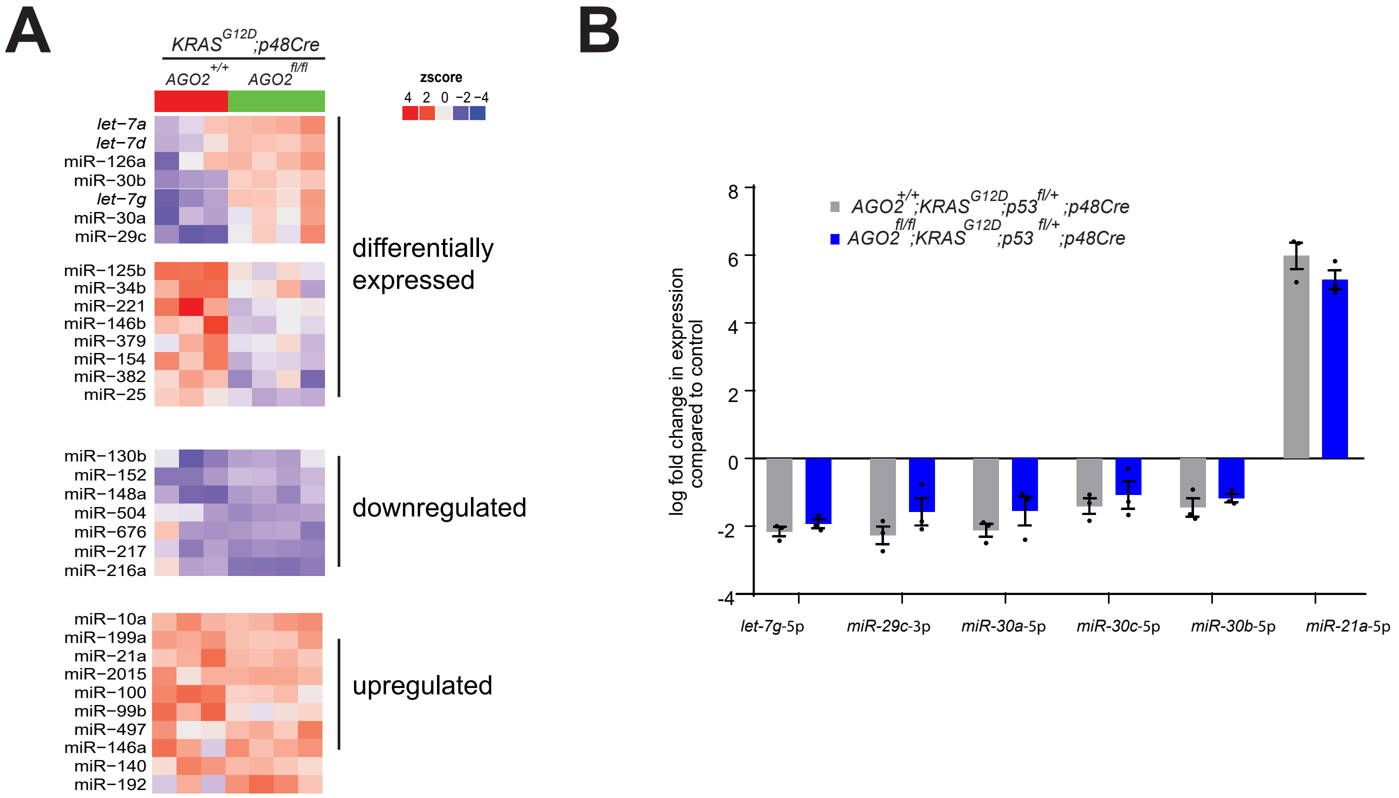

Supplementary Figure 10: microRNA expression changes due to AGO2 loss. (A) Heatmap analysis of the top 50 differentially expressed, upregulated, and downregulated microRNAs in the indicated genotypes at the 500-day time point. microRNAs were quantitated using Exiqon qPCR. Log-transformed miRNA measurements were then centered on mean and divided by standard deviation across samples (z-score). Heatmaps were plotted using the R-package Complex Heatmap. (B) Relative quantitation of select microRNAs in pancreatic tissue obtained from three mice each from the indicated genotypes of the KPC model. In A and B, expression was relative to expression of these microRNAs in pancreatic tissue obtained from mice expressing *p48Cre* alone. Data are presented as mean values +/- SEM.

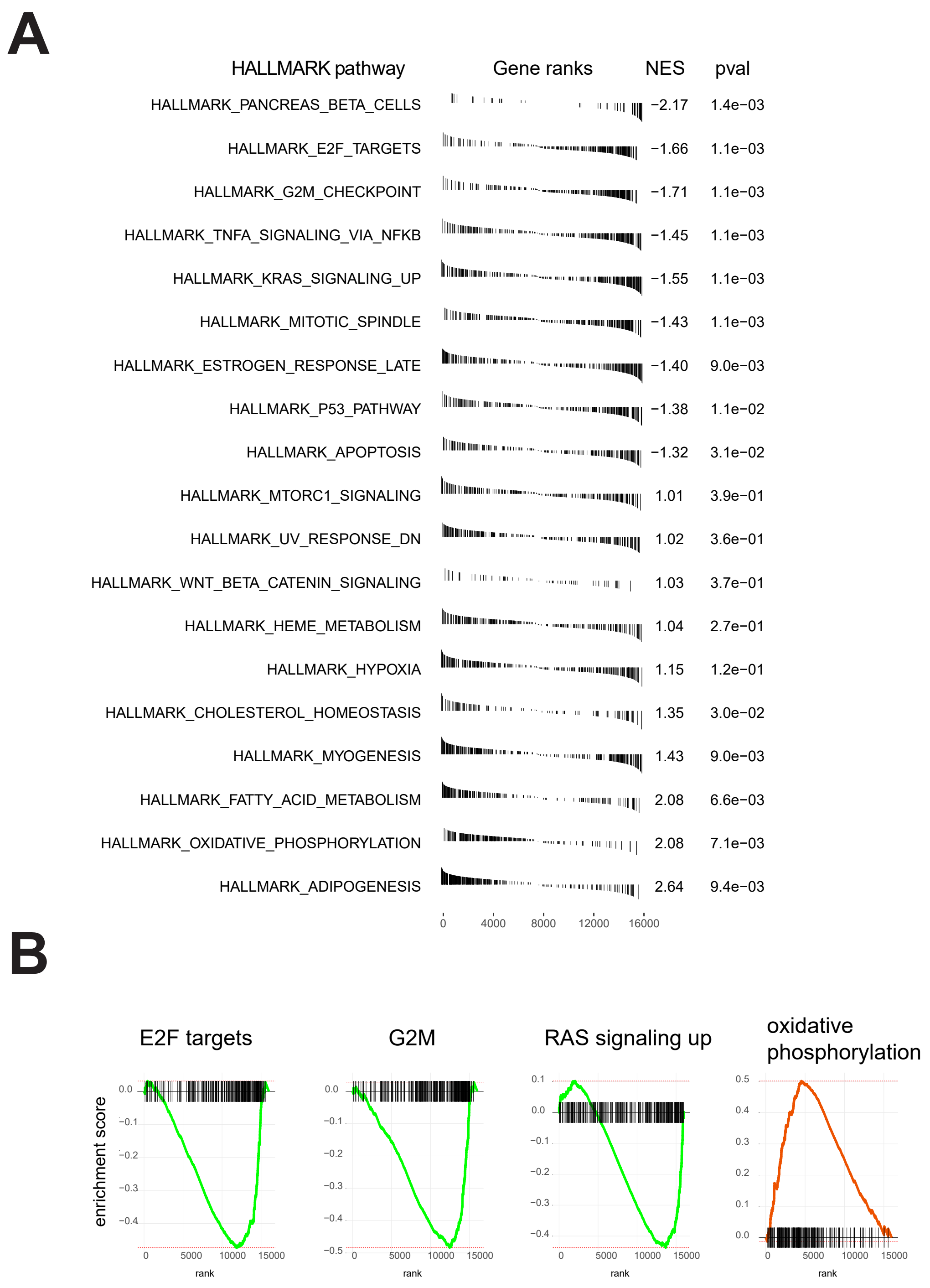

Supplementary Figure 11: Block in PDAC progression due to *AGO2* loss is associated with significantly altered transcriptome. (A) GSEA analysis for all the significantly altered HALLMARK gene sets in pancreatic tissue of *AGO2*$^{fl/fl}$;*KRAS*^{G12D};*p48Cre* mice compared to *AGO2*$^{+/+}$; *KRAS*^{G12D};*p48Cre* mice. (B) GSEA plots for select data sets.

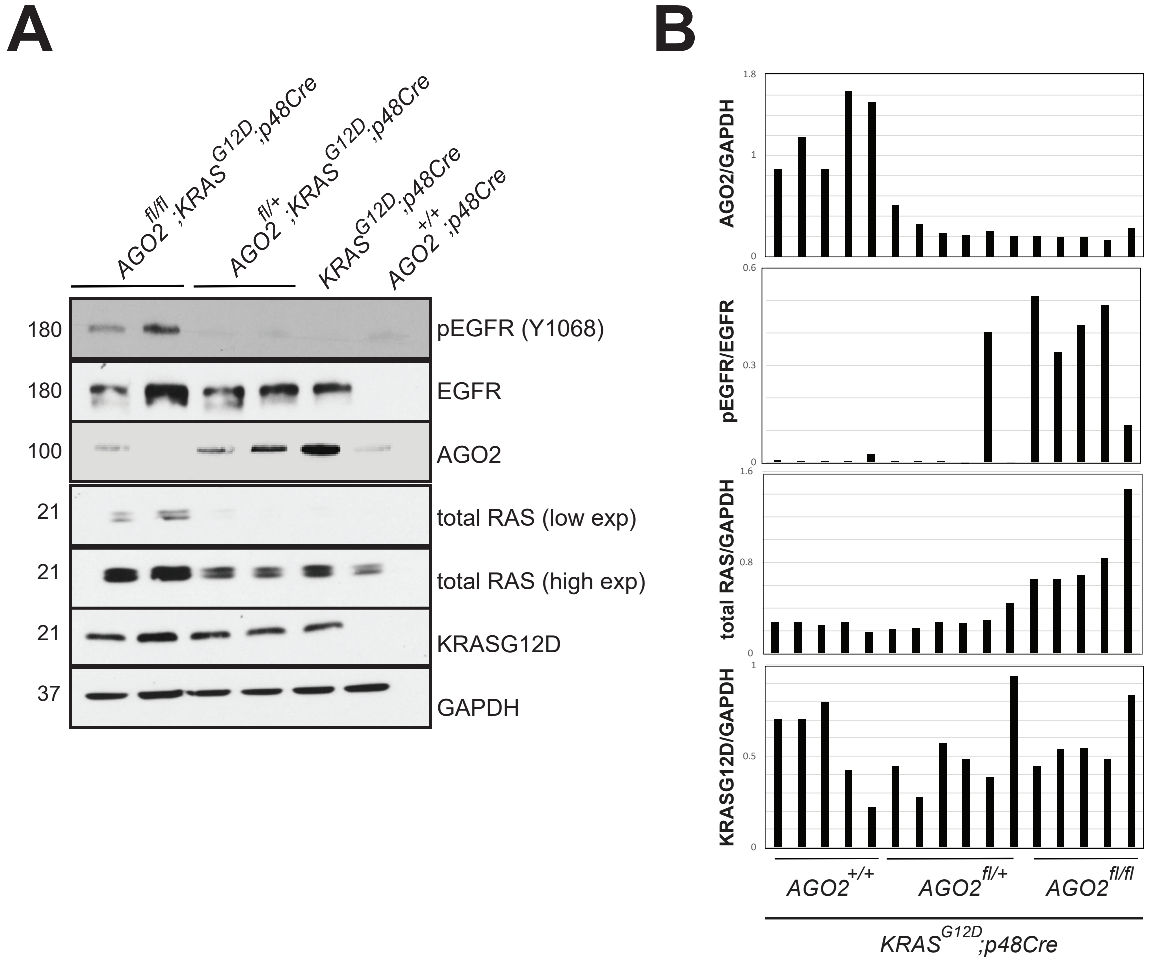

Supplementary Figure 12: AGO2 loss activates EGFR/wild-type RAS signaling. Immunoblot analysis (A) and quantitation (B) of AGO2 and associated RAS signaling molecules from individual pancreas obtained from 12 week old mice (16 individual mice of the indicated genotypes). ImageJ software was employed, and quantitation and arbitrary values were determined with respect to GAPDH expression in the sample. For pEGFR, EGFR expression was used for comparison instead of GAPDH. Numbers on the left of the blots in (A) are protein molecular weights in kDa.

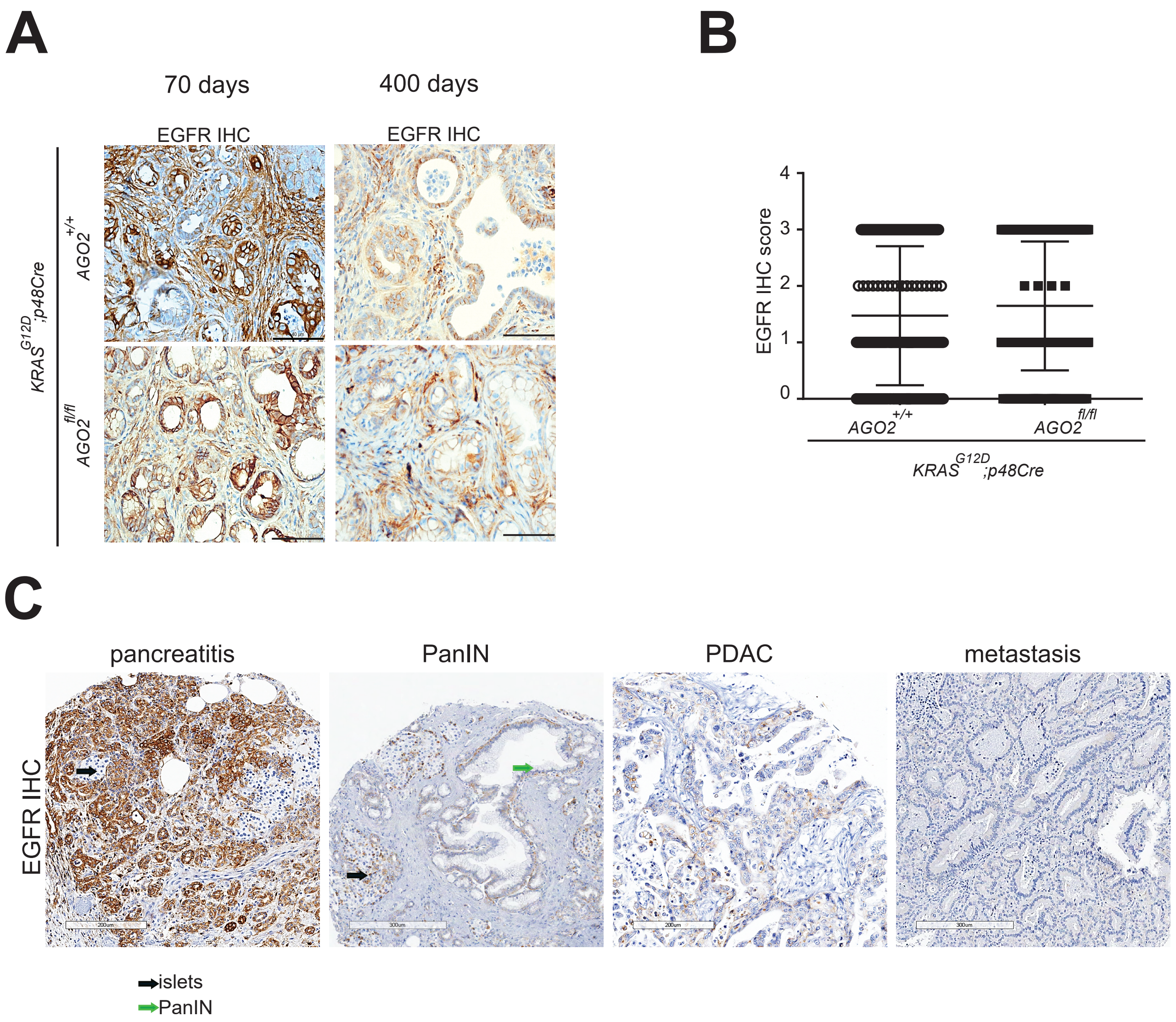

Supplementary Figure 13: EGFR expression is reduced during pancreatic cancer progression. (A) Representative images of EGFR IHC of pancreatic tissue from *AGO2*$^{+/+}$*;KRAS*^{G12D}*;p48Cre* and *AGO2*$^{fl/fl}$*;KRAS*^{G12D}*;p48Cre* mice at 70 or 400-day time points. Scale bar is 100µm. (B) Box plot quantitating total EGFR levels in pancreas obtained from the indicated genotypes at 70-day time point. 300 PanINs from three mice pancreata were assessed for EGFR expression, and intensity was given a value between 0-3, where 0 is considered no expression and 3 is high expression. EGFR expression score= Intensity of EGFR expression* number of PanINs. Two tailed t-test was performed and p value was determined to be 0.072. Data are presented as mean values +/- SEM. (C) Representative images of EGFR IHC on a human pancreatic tissue microarray through disease progression. Scale bar is 300 µm.

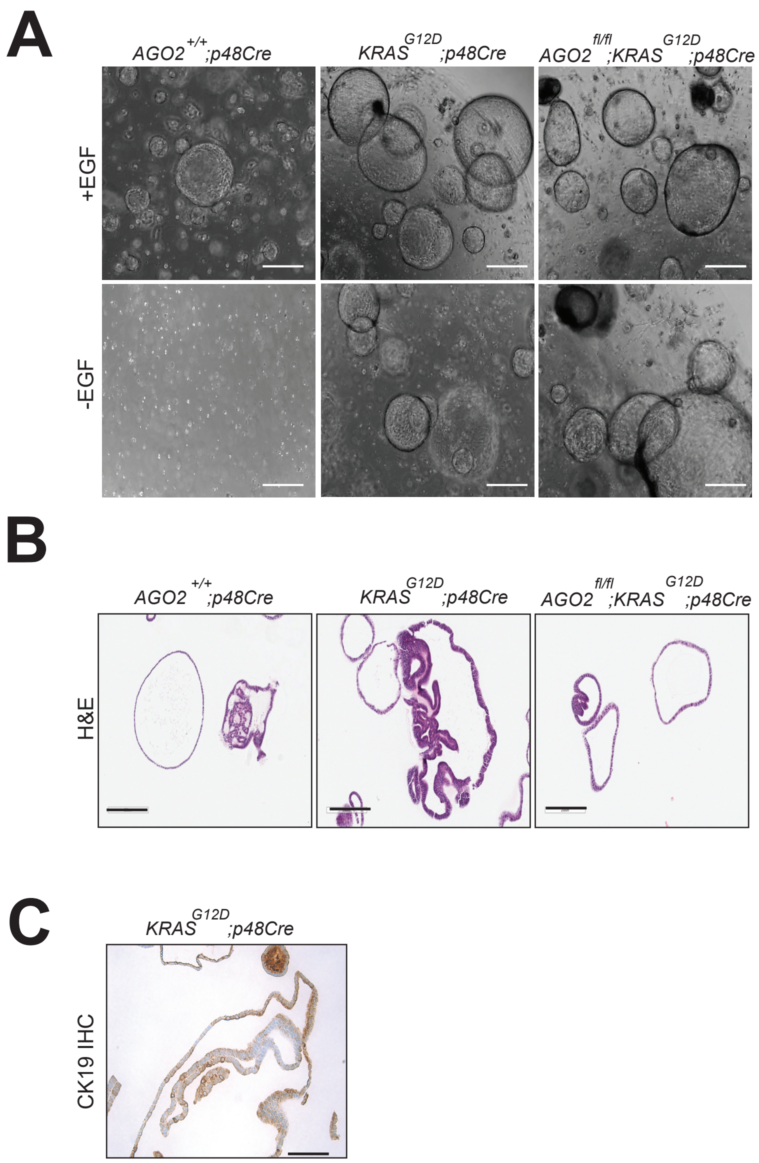

Supplementary Figure 14: Characterization of pancreatic ductal organoids obtained from 12 week old *AGO2$^{+/+}$;KRAS^{G12D};p48Cre* and *AGO2$^{fl/fl}$;KRAS^{G12D};p48Cre* mice. (A) Light microscopic images of mouse pancreatic ductal organoids obtained from the indicated genotypes and cultured in the presence or absence of EGF. (B) H&E staining of pancreatic ductal organoids. Scale bar, 200 μm. (C) CK19 (ductal marker) IHC staining of pancreatic ducts isolated from *KRAS^{G12D};p48Cre* mouse. Scale bar, 100 μm.

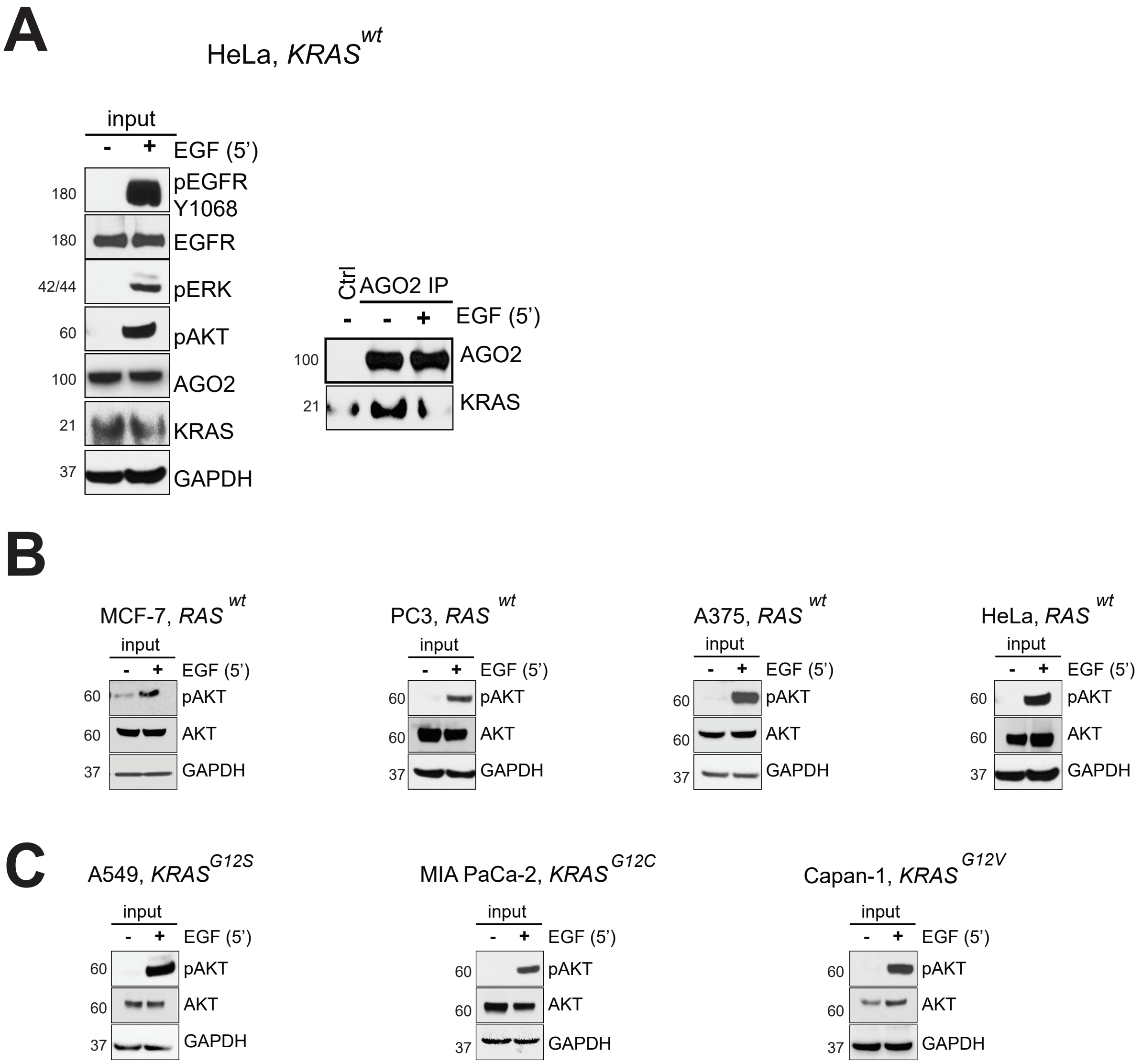

Supplementary Figure 15: EGF stimulation disrupts wild-type KRAS-AGO2 interaction. (A) Immunoprecipitation (IP) of endogenous AGO2 upon EGF stimulation (5'), in HeLa (*KRAS*^{wt}) cells followed by immunoblot analysis of KRAS. Input blots on the left show MAPK activation and levels of various proteins. Input blots show Akt activation upon EGF stimulation in *KRAS*^{wt} cells (B) or *KRAS*^{mt} cells (C) assessed in **Figure 7**. Numbers on the left of the immunoblots in this figure are protein molecular weights in kDa.

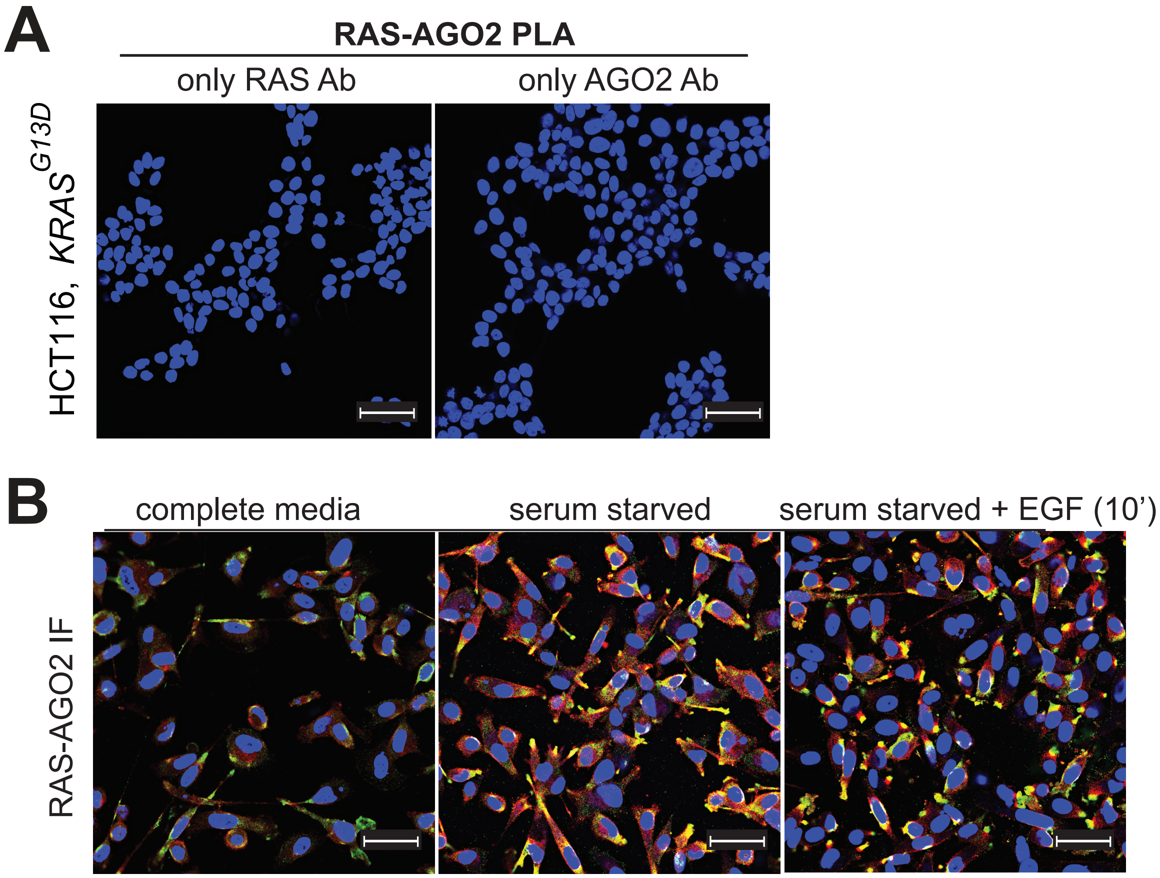

Supplementary Figure 16: Validation of RAS-AGO2 PLA in cell line models. (A) Control RAS-AGO2 PLA performed on HCT116 cells using either RAS or AGO2 antibody. (B) Representative images of RAS/AGO2 IF in PC3 (*KRAS*^{wt}) cells grown under indicated conditions. Confocal images were taken under identical settings across all cell culture conditions. Scale bar, 50 µm.

Supplementary Table 1: Antibodies used in the study

#	Antibody	Vendor	Catalog Number	Application	Specificity
1	Anti-Ras clone 10 (RAS10)	Millipore	05-516	IP (4ug), IB (1:1000), PLA (1:250)	human, mouse
2	K-Ras-2B Antibody (C-19)	Santa Cruz	sc-521	IP (4ug)	human
3	K-Ras monoclonal Ab	Santa Cruz	sc-30	IB (1:200)	human, mouse
4	RAS (G12D Mutant Specific) DH87	Cell Signaling	14429S	IB (1:1000)	human
5	KRAS (3B10-2F2)	Sigma- aldrich	WH0003845M1	IB (1:1000)	Human
6	HRAS- specific Antibody	Proteintech	18295-1-AP	IB (1:1000)	human, mouse, rat
7	AGO2, 11A9	Sigma	SAB4200085	IP (10ug) ,IB (1:1000)	human
8	AGO2 EIF2C2	Sino Biologicals	50683-R036	IB (1:2000), IHC (1:100), PLA (1:200)	human
9	Anti- EGFR (phospho Y1092)	Abcam	ab40815 (EP774Y)	IHC (1:100)	human, mouse
10	Phospho-EGF Receptor (Y1068)	Cell Signaling	2234S	IB (1:1000)	human, mouse
11	Phospho-p44/42 MAPK (Erk 1/2)	Cell Signaling	4376	IB (1:1000)	human, mouse
12	ERK1(K-23)	Santa Cruz	sc-94	IB (1:500)	human, mouse, rat
13	Total p44/42 MAPK (Erk 1/2)	Cell Signaling	9102	IB (1:1000)	human, mouse
14	EGFR Antibody	Abcam	ab52894 (EP38Y)	IB (1:1000),IHC (1:50)	human, mouse, rat
15	EGFR(1005)	Santa Cruz	sc-03	IB (1:500)	human, mouse, rat
16	Anti-FLAG antibody	Sigma	F7425	IP (4ug), IB (1:1000)	human, mouse
17	Phospho-Akt S473	Cell Signaling	4060S	IB (1:1000)	human,mouse
18	AKT (pan) - C67E7	Cell Signaling	4691	IB (1:1000)	human,mouse
19	Anti-Cytokeratin 19 antibody	Abcam	ab133496	IHC (1:1000)	mouse

20	E-cadherin (36)	Ventana Roche	790-4497	IF (1:100)	mouse
21	Nk1.1 (PK136)	Invitrogen	MA1-70100	IHC (1:100)	mouse
22	anti-CD4 (SP35)	Ventana	790-4423	IHC (1:100)	mouse
23	anti-CD68 (KP-1)	Ventana	790-2931	IHC (1:100)	mouse
24	anti-CD8 (SP57)	Ventana	790-4460	IHC (1:100)	mouse
25	Anti-Cytokeratin 19 antibody	Abcam	ab133496	IHC (1:500)	mouse
26	GAPDH- HRP	Cell Signaling	3683	IB (1:5000)	human, mouse
27	Normal mouse IgG	Santa Cruz	sc-2025	Isotype Control IP	mouse
28	Normal Rat IgG	Abcam	ab18450	Isotype Control IP	rat
29	Normal Rabbit IgG	Millipore	12-370	Isotype Control IP	rabbit

IP: Immunoprecipitation; IB: Immunoblot; IHC: Immunohistochemistry; PLA: Proximity Ligation Assay

Validation of the antibodies used in the study:

References and links cited below show specificity and use of the respective antibody for various applications.

1. RAS antibodies (Anti-Ras clone 10 (RAS10), K-Ras-2B Antibody (C-19), K-Ras monoclonal Ab, RAS (G12D Mutant Specific) ,DH87 KRAS (3B10-2F2), HRAS- specific antibodies have been validated[1]. Manufactureres' instruction provides evidence for use of RAS10 in IHC/IF. This is in addition to the extensive validation performed in this study. For all other antibodies, further details are available at the manufacturers' website.
2. AGO2, 11A9 (SAB4200085) *https://www.sigmaaldrich.com/catalog/product/mm/mabe253?lang=en&region=US*
3. AGO2 Sino biological (50683-R036) https://www.sinobiological.com/antibodies/mouse-argonaute-2-50683-r036
4. Anti- EGFR (phospho Y1092) ab40815 (EP774Y) *https://www.abcam.com/egfr-phospho-y1068-antibody-ep774y-ab40815.html* and confirmed in house by immunoblot analysis; also referenced here[2].
5. Phospho-EGF Receptor (Y1068) CST 2234S https://www.cellsignal.com/products/primary-antibodies/phospho-egf-receptor-tyr1068-antibody/2234
6. Phospho-p44/42 MAPK (Erk 1/2) CST 4376 *https://www.cellsignal.com/products/primary-antibodies/phospho-p44-42-mapk-erk1-2-thr202-tyr204-d13-14-4e-xp-rabbit-mab/4370?site-search-type=Products*
7. ERK1(K-23) sc-94 https://www.scbt.com/p/erk-1-antibody-k-23 also referenced[3]
8. Total p44/42 MAPK (Erk 1/2) CST 9102 https://www.cellsignal.com/products/primary-antibodies/p44-42-mapk-erk1-2-antibody/9102

9. EGFR Antibody ab52894 (EP38Y) https://www.abcam.com/egfr-antibody-ep38y-ab52894.html in house validation showed that the IHC pattern of expression for this antibody was identical to EFGR 1005 sc-03. Recognizes single band of correct molecular weight in mouse tissues as well.
10. EGFR(1005) sc-03 https://www.scbt.com/p/egfr-antibody-1005
11. Anti-FLAG antibody Sigma F7425 https://www.sigmaaldrich.com/catalog/product/sigma/f7425?lang=en&region=US
12. Phospho-Akt CST 4060S https://www.cellsignal.com/products/primary-antibodies/phospho-akt-ser473-d9e-xp-rabbit-mab/4060
13. AKT (pan) - C67E7 4691 https://www.cellsignal.com/products/primary-antibodies/akt-pan-c67e7-rabbit-mab/4691
14. Anti-Cytokeratin 19 antibody ab133496 https://www.abcam.com/cytokeratin-19-antibody-epncir127b-ab133496.html
15. E-cadherin (36) Ventana Roche 790-4497 http://reagent-catalog.roche.com/product/1518?type=1958
16. Nk1.1 (PK136) Invitrogen MA1-70100 https://www.thermofisher.com/antibody/product/NK1-1-Antibody-clone-PK136-Monoclonal/MA1-70100
17. anti-CD4 (SP35) Ventana 790-4423 http://reagent-catalog.roche.com/product/31?type=26
18. anti-CD68 (KP-1) Ventana 790-2931http://reagent-catalog.roche.com/product/53?type=48
19. anti-CD8 (SP57) Ventana 790-4460 http://reagent-catalog.roche.com/product/33?type=28
20. GAPDH- HRP CST 3683 https://www.cellsignal.com/products/antibody-conjugates/gapdh-14c10-rabbit-mab-hrp-conjugate/3683
21. Normal mouse IgG sc-2025 https://www.scbt.com/p/normal-mouse-igg
22. Normal Rat IgG Abcam ab18450 https://www.abcam.com/rat-igg2a-kappa-monoclonal-rtk2758-isotype-control-low-endotoxin-azide-free-ab18450.html
23. Normal Rabbit IgG Millipore 12-370https://www.emdmillipore.com/US/en/product/Normal-Rabbit-IgG,MM_NF-12-370

1. Waters, A.M., *et al.* Evaluation of the selectivity and sensitivity of isoform- and mutation-specific RAS antibodies. *Sci Signal* **10**(2017).
2. Wodziak, D., Dong, A., Basin, M.F. & Lowe, A.W. Anterior Gradient 2 (AGR2) Induced Epidermal Growth Factor Receptor (EGFR) Signaling Is Essential for Murine Pancreatitis-Associated Tissue Regeneration. *PLoS One* **11**, e0164968 (2016).
3. Navas, C., *et al.* EGF receptor signaling is essential for k-ras oncogene-driven pancreatic ductal adenocarcinoma. *Cancer Cell* **22**, 318-330 (2012).

Supplementary Table 2:

Cases in *KRAS*^{G12D};*AGO2fl/fl;p48Cre* cohort with abnormal pathologies or death prior to 500 day time point

Ear tag#	3331	2430	2385
Age (days)	368	500	500
Liver			
Ascites			
Lungs			
Diaphragm			
Hemorrhage			
Lymph nodes			
Kidneys			
Jaundice			
Enlarged Spleen			
**Pathological Evaluation**			
PDAC	no anaplastic PDAC or PDAC	no PDAC	no PDAC
Pancreas	low grade PanINs and cyst	only low grade PanIN	only low grade PanIN
AGO2 expression in pancreas	No AGO2 expression in the cyst or PanINs	no AGO2 expression in early PanINs	no AGO2 expression in early PanINs
Pancreatic Cyst	Yes	No cyst	No cyst
Further comment/ other pathology	resembles mucinous cystic neoplasm	likely benign lesion in lung, unknown origin	likely benign lesion in lung, unknown origin
AGO2 expression in mets?	n/a	AGO2 expressed in benign lung lesion	AGO2 expressed in benign lung lesion
Cause of Death	unknown	not dead before 500 days	not dead before 500 days

Supplementary Table 3: Primers used in the study

I)

Transcript	Primer Sequence
m*HRAS*_Fwd	*5' GCTTCCTCTGTGTATTTGCCA 3'*
m*HRAS*_Rev	*5' CTTTCACCCGCTTGATCTGC 3'*
m*KRAS*_Fwd	*5' GTTAGCTCCAGTGCCCCAAT 3'*
m*KRAS*_Rev	*5' ATTCCCTAGGTCAGCGCAAC 3'*
m*NRAS*_Fwd	*5'ACTGGCCAAGAGTTACGGAA 3'*
m*NRAS*_Rev	*5' TGGCGTATCTCCCTTACCAG 3'*
m*AGO2*_Fwd	*5'GATCGCCAAGAGGAGATCAG3'*
m*AGO2*_Rev	*5'GCCTCCCAGTTTGACATTGA3'*
m*GAPDH*_Fwd	*5' AAGGTCATCCCAGAGCTGAA 3'*
m*GAPDH*_Rev	*5' CTGCTTCACCACCTTCTTGA 3'*
hAGO2 ORF Fwd	*5' ATCAACGTCAAGCTGGGAGG 3'*
hAGO2 ORF Rev	*5' GTGACGTCTGCTCCCAGAAA 3'*

II) *KRAS*^{G12D} conditional PCR primers 1) 5' *gtc ttt ccc cag cac agt gc* 3', 2) 5' *ctc ttg cct acg cca cca gct c* 3', and 3) 5' *agc tag cca cca tgg ctt gag taa gtc tgc a* 3' according to the Tyler Jacks lab protocol described in detail at

https://jacks-lab.mit.edu/protocols/genotyping/kras_cond

III) Primers used to generate Y393F

hAGO2_Y393F_Fwd 5'*AAATTCACGGACGAATGGATCTGTGTTGAAACTTGCAC*3' and
hAGO2_Y393F_Rev 5'*GTGCAAGTTTCAACACAGATCCATTCGTCCGTGAATTT*3'.

Supplementary Table 4: Cell lines used in the study

Number	Cell Line	Source	Tissue	Type	***KRAS* or *AGO2* status**	From
1	PC3	Human	Prostate	Cancer	*KRAS* WT	ATCC
2	MCF7	Human	Breast	Cancer	*KRAS* WT	ATCC
3	A375	Human	Melanoma	Cancer	*KRAS* WT	ATCC
4	A549	Human	Lung	Cancer	*KRAS* G12S	ATCC
5	MIA PaCa-2	Human	Pancreas	Cancer	*KRAS* G12C	ATCC
6	CAPAN-1	Human	Pancreas	Cancer	*KRAS* G12V	ATCC
7	DLD-1 (MUT/WT)	Human	Colorectal	Cancer	*KRAS* G13D	ATCC
8	H358	Human	Lung	Cancer	*KRAS* G12C	ATCC
9	HCT116	Human	Colorectal	Cancer	*KRAS* G13D	ATCC
10	PANC-1	Human	Pancreas	Cancer	*KRAS* G12D	ATCC
11	Panc 05.04	Human	Pancreas	Cancer	*KRAS* G12C	ATCC
12	Panc 10.05	Human	Pancreas	Cancer	*KRAS* G12C	ATCC
13	HEK293FT	Human	Embryonic kidney	Benign	*KRAS* WT	ATCC
14	MEF,parental	Mouse	Embryonic fibroblast	Benign	*KRAS* WT	Zamore lab (PMID: 21878547)
15	*AGO2* MEF-/-	Mouse	Embryonic fibroblast	Benign	*AGO2* knockout	Zamore lab (PMID: 21878547)
16	*AGO2* MEF-/-+*AGO2*	Mouse	Embryonic fibroblast	Benign	*AGO2* knockout overexpressing *AGO2*	Zamore lab (PMID: 21878547)
17	Rasless MEFs	Mouse	Embryonic fibroblast	Benign	RAS deficient	The RAS Initiative https://www.cancer.gov/research/key-initiatives/ras/outreach/reference-reagents
18	HEK293T-Rspo1-Fc	Human	Embryonic kidney	Benign	R-spondin expressing	Calvin Kuo, Stanford University, USA
19	HEK293T-Noggin	Human	Embryonic kidney	Benign	Noggin expressing	Gis van den Brink, Tytgat University, Netherlands
